# Use of Prophylactic Mesh When Creating a Colostomy Does Not Prevent Parastomal Hernia: A Randomized Controlled Trial—STOMAMESH

**DOI:** 10.1097/SLA.0000000000002542

**Published:** 2018-10-23

**Authors:** Christoffer Odensten, Karin Strigård, Jörgen Rutegård, Michael Dahlberg, Ulrika Ståhle, Ulf Gunnarsson, Pia Näsvall

**Affiliations:** ∗ Department of Surgical and Perioperative Sciences, Umeå University, Luleå, Sweden; † Sunderby Research Unit, Umeå University, Luleå, Sweden; ‡ Department of Imaging and Physiology, Karolinska University Hospital, Luleå, Sweden.

**Keywords:** mesh, parastomal hernia, prophylaxis

## Abstract

**Objective:**

The aim of this study was to determine whether parastomal hernia (PSH) rate can be reduced by using synthetic mesh in the sublay position when constructing permanent end colostomy. The secondary aim was to investigate possible side-effects of the mesh.

**Background:**

Prevention of PSH is important as it often causes discomfort and leakage from stoma dressing. Different methods of prevention have been tried, including several mesh techniques. The incidence of PSH is high; up to 78%.

**Methods:**

Randomized controlled double-blinded multicenter trial. Patients undergoing open colorectal surgery, including creation of a permanent end colostomy, were randomized into 2 groups, with and without mesh. A lightweight polypropylene mesh was placed around the colostomy in the sublay position. Follow up after 1 month and 1 year. Computerized tomography and clinical examination were used to detect PSH at the 1-year follow up. Data were analyzed on an intention-to-treat basis.

**Results:**

After 1 year, 211 of 232 patients underwent clinical examination and 198 radiologic assessments. Operation time was 36 minutes longer in the mesh arm. No difference in rate of PSH was revealed in the analyses of clinical (*P* = 0.866) and radiologic (*P* = 0.748) data. There was no significant difference in perioperative complications.

**Conclusions:**

The use of reinforcing mesh does not alter the rate of PSH. No difference in complication rate was seen between the 2 arms. Based on these results, the prophylactic use of mesh to prevent PSH cannot be recommended.

Parastomal hernia (PSH) is a common complication after stomal surgery. Though the exact incidence has not been fully established, figures range between a few and 78%.^1–3^ Most PSHs develop within 2 years of surgery but can occur up to 30 years later. Approximately, one third of patients operated for rectal cancer in Sweden have a permanent colostomy, and 24% to 39% of patients with a “temporary” stoma never have it reversed.^4–6^ PSH causes difficulties with stoma dressing and leakage, increases the risk for incarceration, and has a negative impact on the patient's quality of life.^7–9^

A number of strategies have been proposed to prevent the formation of PSH after primary surgery: choice of stoma placement through versus lateral to the rectus sheath, transperitoneal versus extraperitoneal, and correct sizing of the trephine.^2,10^ None of these seems to reduce the incidence of PSH. Furthermore reported 30-day morbidity and mortality rates of planned repair procedures are 8% to 36% and 0% to 5%, respectively ^11–13^ Emergency PSH repair has a reported mortality rate of 11% to 25%.^14,15^ The development of stoma techniques that reduce the risk for PSH is thus a field of research that should be given priority.

Placement of a mesh to prevent PSH formation has been proposed. Eight small and 2 large randomized controlled trials (RCT) have been published during the past 2 decades. Pooled data from these studies show promising results,^16^ though mesh location, choice of mesh material and surgical approach varied.^17–23^ The use of mesh did not increase complication rate, but none of the trials was designed to assess complication. On the contrary, 1 retrospective trial comparing PSH rate before and after the introduction of routine prophylactic mesh around the stoma, at a unit that repeatedly produced top results in the Swedish Rectal Cancer Registry, showed no difference in complication rates between groups.^24^ The Swedish National Board of Health and Welfare classifies the use of prophylactic mesh around stomas as, “Research and Development,” because of the weak scientific evidence; larger RCTs comparing prophylactic mesh with no mesh are required.^25^

In view of this uncertainty, we designed a double-blinded multicenter RCT to evaluate the effects of using a prophylactic mesh around the stoma. The primary aim was to evaluate the PSH rate in both groups, and a secondary aim was to assess the risk for early complications. Our hypothesis was that polypropylene mesh in the sublay position around a colostomy decreases the risk for PSH.

## METHODS

### Study Design

The study was designed as a double-blinded multicenter RCT. Patients were randomized to 2 groups, those with and those without prophylactic mesh around the stoma. The study protocol adhered to the Helsinki Declaration and was approved by the Regional Ethics Committee at Umeå University, Sweden (DNR 07-081 M). The study was registered at ClinicalTrials.gov (Identifier: NCT00917995) and adhered to the CONSORT 2010 criteria for RCTs.

### Patients

All patients scheduled for permanent colostomy at one of the participating hospitals and who met the inclusion criteria (no previous stoma; older than 18 years; and with informed consent), were eligible to participate in the study. Exclusion criteria were: expected survival less than 3 years; fecal peritonitis; previous stoma; and no informed consent.

### Surgery

Colostomy without mesh was taken through the rectus muscle, at a site marked by a stoma therapist before surgery. The height of the stomal nipple was at least 1 cm, and neither intraabdominal lateral closure nor fixation to the fascia was performed. Mucocutaneous interrupted monofilament sutures were used to fix the stoma to the skin.

Colostomy with mesh incorporated a lightweight polypropylene mesh (density 25–40 g/m^2^) in the sublay position. A 10 × 10 cm space for the mesh was created dorsal to the rectus abdominis muscle but anterior to the posterior rectus sheath and peritoneum. The bowel was passed through the rectus muscle via a cruciform incision in the middle of the mesh. Single nonabsorbable monofilament sutures anchored the mesh laterally in the pocket. Medially the mesh was incorporated in the running suture closing the fascia. A potential risk is that the mesh would not reach the midline; however, no such effect was described in the original article.^26^ The surgical technique used had been described in an earlier study comparing stoma with and without prophylactic mesh.^26^ An instruction video, made by Israelsson et al at the Department of Surgery, Sundsvall, was distributed to all participating centers to ensure that a uniform surgical technique was used. Fixation to the skin was performed with the same technique as when creating a colostomy without mesh. Surgery was performed by an experienced colorectal surgeon with an annual volume of at least 100 major surgical procedures.

Postoperative mobilization was according to each hospital's routine.

### Study Outcome

Early complications were evaluated at 1 month, and late complications and possible recurrence of parastomal hernia were assessed at a 1-year follow up. The examiner was blinded to the group the patient was randomized to. Primary endpoint was the rate of parastomal hernia in both groups, judged clinically and by computerized tomography (CT) 1 year after surgery. Clinical examination was performed in both upright and supine positions by digital palpation within the stoma at rest and during the Valsalva maneuver. Findings were classified as a bulge or PSH. Definition of a bulge was a protrusion around the stoma noticed by the surgeon and/or the patient but deemed not to be a hernia clinically. CT was performed in the supine position, with or without intravenous contrast; the Valsalva maneuver was not performed. All CT examinations were evaluated by a radiologist with a special interest in parastomal hernia. The findings were classified according to the Moreno-Matias scale, classified as validated by the European Hernia Society, based on the content of the hernia: no hernia = 0; bowel forming the stoma with hernia sac < 5 cm = IA; bowel forming the stoma with hernia sac > 5 cm = IB; hernia sac containing omentum = II; and hernia sac containing loop other than bowel forming the stoma = III.^27,28^

### Sample Size

As the vast majority of patients receiving a colostomy are operated for colorectal cancer, a 5-year survival rate around 50% is likely. Assuming a parastomal hernia incidence of 20% ^7^ without and 5% with mesh, a 5-year survival rate of just over 50%, and a significance level of 95%, it would require 220 patients to achieve a power of 80%.

### Randomization and Blinding

Randomization was performed using sealed envelopes, stratified per hospital in blocks of four, to ensure balance between the 2 arms. The envelopes were prepared by the Regional Cancer Center North, Umeå University Hospital, in bundles of 100 per hospital, without the help of the investigators. Neither the patient nor the surgeon assessing the patient postoperatively was informed on which arm the patient had been randomized to. Postoperative assessment was made by a surgeon not involved in the primary procedure. If randomization of a patient was performed incorrectly, the patient was replaced by 3 new patients to ensure maintenance of power.

### Data Collection

Data were collected prospectively and patients were followed by protocol with individual case report forms (CRF). All data were entered into an Excel-database.

After completion of inclusion, a separate analysis was performed to describe the cohort of patients who met the inclusion criteria but were not included in the study. To obtain a representative group of “not included” patients, all other colostomy operations performed during the inclusion/trial period at Umeå University Hospital, Sunderby Regional Hospital and Mora County Hospital were assessed for demographic data and reasons for not being included.

### Statistical Analysis

The IBM SPSS Statistics 24 software program was used. All analyses of group variables were performed with the *χ*^*2*^ test and all analyses of continuous variables were performed with the independent Student *t* test; a *P*< 0.05 was considered significant. The population was tested for skewness and kurtosis (measure describing outliers in a data set) using SPSS Statistics. As normal distribution was shown, parametric statistics were used.

## RESULTS

Eight hospitals participated in the study: 4 university hospitals (Karolinska University Hospitals in Huddinge and Solna, Stockholm, Uppsala University Hospital, and Umeå University hospital); 2 regional hospitals (Sunderby Regional Hospital and Helsingborg Regional Hospital); and 2 county hospitals (Mora County Hospital and Nyköping County Hospital). The total catchment area of the 8 hospitals is approximately 1,475,000 individuals; approximately 15% of the Swedish population.

**FIGURE 1:**
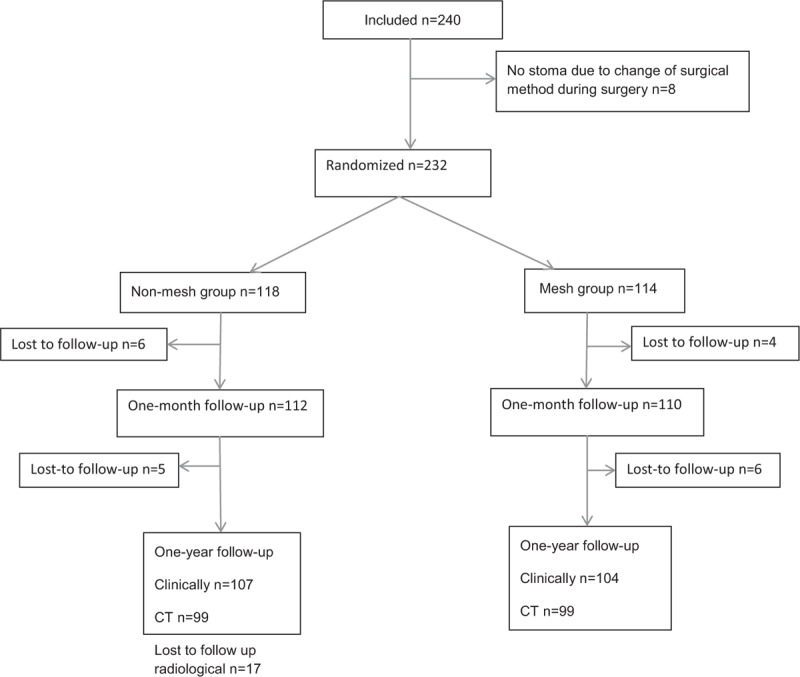
Consort diagram.

From December 2007 through October 2015, 240 patients were enrolled in the trial (Fig. 1). All operations were planned and performed according to a standard protocol within the National Health Service. Eight patients were enrolled in the study but had to be excluded because of change in surgical approach during the operation. Seven of these patients had been excluded and 21 new patients included and randomized according to the study design. Unfortunately, when all CRFs had reached the study center, one more patient had to be excluded, and could not be replaced as the trial had been closed.

There was no significant difference in baseline data between the mesh group and the nonmesh group (Table 1) apart from duration of surgery, which was significantly longer in the mesh group (*P =* 0.019).

**TABLE 1:** Basic Demographic Data.

	Nonmesh Group n = 118	Mesh Group n = 114	*P*
Age, yrs, range	69.9 (35–89)	69.7 (41–86)	0.877
Sex: male	62 (52%)	74 (65%)	0.056
ASA 1+2^*^	78 (70%)	82 (74%)	0.578
ASA 3^*^	34 (29%)	28 (26%)	0.578
Smoker^†^	8 (7%)	12 (11%)	0.326
BMI, range	26.3 (18.5–43.7)	26.1 (16.7–37.8)	0.766
Cancer	106 (90%)	106 (93%)	0.392
Operation time, mins, range	287 (84–625)	323 (70–616)	0.019
Planned surgery	118 (100%)	113 (99%)	0.308
Length of stay, d, range	12 (2–44)	12 (4–44)	0.792

Basic Demographic Data in the Trial Population.

^*^ASA-class was stated in 222 patients.

^†^Smoking habit was stated in 216 patients. Median values are shown for: age, BMI, operation time and length of stay.

Comparison of patients in the “not included” group, that is, meeting the inclusion criteria but not included, with those in the STOMAMESH trial group, showed significantly lower proportions undergoing emergency surgery (*P* < 0.001) and with ASA-class 3 (*P* < 0.001) in the study group. Furthermore, patients in the study population were older (*P =* 0.010) (Table 2).

**TABLE 2:** Study population vs “Not included”.

	STOMAMESH Population n = 232	Not Included n = 434	*P*
Age, yrs, range	69.8	67.5	0.010
ASA 1+2	167 (71.9%)	229 (52.8%)	<0.001
ASA 3	65 (28.1%)	205 (47.2%)	<0.001
Sex: male	135 (58.0%)	217 (50%)	0.020
Planned surgery	231 (99.6%)	337 (77.6%)	<0.001

The group of patients included in the STOMAMESH trial compared with the patients who could have been but were “Not included” at 3 hospitals (Sunderby hospital, Umeå University hospital, and Mora hospital) during the inclusion period. Median values are shown for age.

When excluding emergency procedures from both groups differences in age, ASA, and operation time between the 2 groups remained (Table 3).

**TABLE 3:** Study population vs "Not included" emergency cases excluded.

	STOMAMESH Population n = 231	Not Included n = 337	*P*
Age, yrs, range	69.8	67.7	0.043
ASA 1+2	166 (71.9%)	200 (59.3%)	0.002
ASA 3	65 (28.1%)	137 (40.7%)	0.002
Sex: male	135 (58.0%)	164 (48.7%)	0.029

The group of patients included in the STOMAMESH trial compared with the patients who could have been but were “Not included” at 3 hospitals (Sunderby hospital, Umeå University hospital, and Mora hospital) during the inclusion period. Emergency procedures and ASA 4 excluded. Median values are shown for age.

No significant differences were found when data were stratified according to hospital.

After 1 year, 211 of 232 patients included (91%) were followed up clinically, and 198 of 232 patients (85%) were examined with CT (Fig. 1). According to the requisites for the original power calculation, this gives a de facto power of 91%. Reasons for being lost to follow up were: incomplete follow up because of surgical complication (3 patients); progression of disease (1 patient); development of dementia (1 patient); refusal of patient to participate further in the study (9 patients); and death (7 patients). When comparing the mesh and nonmesh groups, there was no significant difference in the rate of bulge (*P* = 0.631), clinically judged PSH (*P* = 0.866), or PSH on the CT (*P* = 0.748) (Table 4). There was no difference in complication rates at 1-month follow up between the groups (Table 5). Reoperations performed during the first postoperative month were because of small bowel obstruction requiring adhesiolysis (1 in each group), conversion to transverse colostomy (1 in the mesh group), revision of the enterostomy (2 in the mesh group and 3 in the nonmesh group), bleeding (2 in the mesh group), superficial/deep infection (1 in each group), and wound rupture (1 in the nonmesh group). A further 2 patients in the nonmesh group and 4 patients in the nonmesh group were reoperated within the first postoperative year. At the 1-year follow up, a total of 8 patients in the nonmesh and 12 in the mesh group had been reoperated.

**TABLE 4:** Hernia rate at one year.

	Nonmesh Group n = 107	Mesh Group n = 104	*P*
Hernia (judged clinically)	32 (30%)	30 (29%)	0.866
Bulge, no hernia (judged clinically)	18 (17%)	15 (14%)	0.631
Hernia classified 2 and 3 on CT	28 (26%)	25 (24%)	0.748
Hernia classified 1, 2, and 3 on CT	36 (34%)	33 (32%)	0.765

Follow Up at 1 Year Evaluating Presence of Parastomal Hernia. CT-scan was performed on 99 patients in each group and the findings were classified according to the model by Moreno-Matias. Bulge was defined as a protrusion around the stoma judged not to be a hernia.

**TABLE 5:** Complications at one month.

	Nonmesh Group n = 112	Mesh Group n = 110	*P*
Overall complications	36 (32%)	38 (34%)	0.668
Surgical complications	31 (28%)	30 (27%)	0.947
Wound infection	16 (14%)	17 (15%)	0.785
Deep infection	9 (8%)	7 (6%)	0.644
Intestinal obstruction	1 (1%)	2 (2%)	0.618^*^
Stoma necrosis	8 (7%)	5 (4%)	0.570^*^
Reoperation within 30 days	6 (5%)	7 (6%)	0.783^*^
Other complications			
Acute myocardial infarction	2 (2%)	2 (2%)	1.000^*^
Pneumonia	1 (1%)	2 (2%)	0.618^*^
Thrombosis	0	2 (2%)	0.242^*^
Urinary tract infection	1 (1%)	5 (4%)	0.116^*^

Complications at the 1-Month Follow Up in the Trial Population.

^*^The assumption for the *χ*^*2*^ test was not fulfilled in these comparisons and Fischer exact test was used.

## DISCUSSION

This trial, the largest multicenter RCT on stomal surgery in routine healthcare, revealed no difference in PSH rate between procedures incorporating a prophylactic mesh around the stoma and those with no mesh. Complication and reoperation rates did not differ between groups. Compared with previously published trials analyzing the use of mesh in stomal surgery, the current trial was based on a larger population and a systematic follow up including both clinical and CT assessment of PSH. Furthermore, this study was multicenter and thus represents the use of mesh in normal clinical practice using well-established routines. It covers most regions in Sweden, and represents approximately 15% of the population. Furthermore, unlike other studies, this study was blinded to both patient and examiner, which could be an explanation for the diverging results.^18–20,26^ This trial also includes different types of hospital and levels of specialization, whereas most previous trials are from single specialist Institutions.^20,22,26,29^ The trial reached a *de facto* power of 91%. The only significant difference found between the 2 treatment arms was a longer operation time (median 38 minutes) for the mesh group corresponding to the time expected for application of the mesh. Interestingly, in a large retrospective study in Sweden including 206 patients, reported from a single institution, it was not possible to show a difference in PSH between procedures with or without mesh.^24^ Follow-up assessment in that trial also included both clinical investigation and CT. That trial was carried out at a high-volume single center with top results in the Swedish Rectal Cancer Registry.^4^ This center did not participate in the STOMAMESH trial, but had similar results.

We also describe the patient group eligible for inclusion but not taking part, the “not included” group, a procedure rarely performed in previous trials.^18,19,21,22,26,29^

A potential weakness of this study is that we choose to investigate the population of “not included” patients at 3 hospitals (Sunderby, Umeå, and Mora), to get a representative sample, instead of doing this at all participating hospitals. Furthermore, the choice to use routine CT might also be considered a weakness. In some studies, the patients were placed in the supine position or performed the Valsalva maneuver during the examination.^30^ This might have increased the sensitivity of CT in this study. The reason not doing this is that abdominal CT scan with the patient in the supine position is standard. An additional CT with Valsalva would have increased radiation exposure and would have demanded extra resources, thus deviating from routine practice. The majority of CT scans were included in routine colorectal cancer follow up at the hospitals participating. It would seem unlikely; however, that this difference in CT follow up should favor one or other of the treatment arms.

The reason for choosing the sublay polypropylene mesh technique was that this had been investigated in a number of previous studies and that laparoscopic surgery had not been accepted as standard practice in Sweden, 2005. Open surgery was thus the natural choice.

Only 1 patient in the STOMAMESH population had emergency surgery whereas 96 (22.4%) in the “not included” population were operated as an emergency. This was a systematic error in the study design but does not entirely explain the differences in ASA and age. One might argue that we should have excluded emergency procedures in the study design. Consequently, we cannot extrapolate the results from elective surgery to those having emergency surgery with a contaminated abdomen.

This study did not investigate or quantify symptoms arising from PSH. It is well known that not all PSHs actually cause symptoms ^7^ and this has been used as an argument against the use of prophylactic mesh. The PSH rate in our study was more than three times that in a recent review article (29.4% vs 8.9%),^16^ implying that results from smaller studies performed at single institutions with a special interest in PSH are difficult to extrapolate to routine clinical practice. The meticulous follow up in this study might well have generated the higher PSH rates. No negative side-effects of prophylactic mesh were detected despite the rather large trial population.

There have been several trials studying PSH, and the majority has shown positive results regardless of which prophylactic mesh is used when forming a colostomy.^16^ These authors recommend the use of mesh when creating a colostomy. The results from the current study do not support this conclusion. Comparing our trial with the Dutch trial,^18^ which was second largest and the one most similar in design to ours, the Dutch trial did not include routine CT follow up and blinding which could explain the difference in results. In view of the dubious need for prophylactic mesh and the divergent trial results to date, we cannot recommend the use of prophylactic mesh when creating a colostomy. Furthermore, we do not have a clear picture of how many patients who develop a PSH suffer symptoms related to their PSH. The most promising results have been reported using mesh for repair of PSH, including the Sugarbaker procedure.^29,31^ Morbidity risk with mesh repair is high and deaths have been reported.^16,32,33^ There are reports of long-term complications from mesh implantation in the form of discomfort, pain, and fistula formation.^33–35^ Should mesh be used prophylactically on a large scale, the incidence of these complications would likely increase. In fact, long-term follow-up studies on the use of prophylactic parastomal mesh are rare indeed.

## CONCLUSIONS

In routine surgical care, no difference in rate of PSH can be seen when creating an elective colostomy with or without a reinforcing mesh. The management of PSH is still a considerable surgical challenge and there is no generally accepted method for its prevention. Further research should focus on developing preventive measures and improving methods for PSH repair.
